# ST-GraphRCA: A Root Cause Analysis Model for Spatio-Temporal Graph Propagation in IoT Edge Computing

**DOI:** 10.3390/s26051474

**Published:** 2026-02-26

**Authors:** Tianyi Su, Ruibing Mo, Yanyu Gong, Haifeng Wang

**Affiliations:** 1 Department of Electrical and Information Engineering, Shandong University of Science and Technology, Jinan 250031, China; 2 School of Information Science and Engineering, Linyi University, Linyi 276012, China; moruibing@lyu.edu.cn

**Keywords:** root cause analysis, edge computing, internet of things, high-utility mining, Dynamic Time Warping, causal inference

## Abstract

Real-time processing demands for massive IoT sensor data necessitate reliance on distributed microservice systems within edge clusters. However, pinpointing the root cause of anomalies within these edge microservice clusters poses a critical challenge for intelligent IoT operation and maintenance. To address the issue, a spatio-temporal graph propagation model ST-GraphRCA is proposed for root cause analysis in IoT edge environments. Our approach begins by resolving the fundamental issue of time-series asynchrony across distributed multi-source metrics. A PCA-DTW hybrid feature extraction method is introduced with a dynamic alignment strategy to mitigate the effects of random network delays and data deformation without requiring prior synchronization. Subsequently, ST-GraphRCA constructs a stream-based forward propagation graph based on the flow conservation principle. By integrating dynamic edge weights with node-level input–output anomaly scores, ST-GraphRCA precisely infers fault propagation pathways and identifies potential root cause candidates through causal reasoning. Finally, a topology-constrained high-utility mining algorithm filters these candidates. Using a constraint matrix, the algorithm filters out unreachable service combinations to locate low-frequency and high-risk root causes. Experimental results indicate that ST-GraphRCA achieves an F1-Score of 0.89, outperforming existing methods. In resource-constrained edge scenarios, its average localization time is merely 238.8 ms, representing a six-fold improvement over key benchmarks. Thus, ST-GraphRCA not only provides an efficient anomaly fault tracing solution for large-scale IoT systems but also offers technical support for the intelligent operation and maintenance of distributed microservice systems.

## 1. Introduction

IoT technology has enabled the massive deployment of smart sensors. These sensors are now deployed in diverse domains including intelligent urban management, industrial automation, precision agriculture and ecosystem monitoring. These smart sensors continuously generate massive amounts of time-series data that must be transmitted to cloud centers for processing [1,2]. However, the network latency associated with cloud computing is unable to meet the ubiquitous computing demands of the IoT. As a novel distributed computing paradigm, edge computing effectively addresses the demand for computing power at the data source [3]. Edge data centers, which comprise clustered edge servers, perform two primary functions as an intermediary between cloud and distributed devices: the local reception and aggregation of sensor data streams, and the deployment and execution of microservice-based applications [4]. By executing localized data preprocessing, analysis, and real-time decision-making tasks via distributed microservices, response latency is significantly reduced, and the load on the core network is alleviated [5].

The migration to edge computing clusters, while enhancing service delivery efficiency, introduces significant system complexity. The complexity originates in the orchestration of numerous distributed services. Specifically, these services are both highly interdependent and require precise coordination. These distributed services process real-time sensor data through complex invocation chains [6,7]. Due to the complexity of the edge environment and limited computing resources, software service anomalies such as response timeouts, throughput degradation or deadlocks occur frequently. These anomalies seriously impact system Quality of Service (QoS) and reliability. Therefore, anomaly detection and root cause analysis for software services are of significant importance [8]. When anomalies are detected in system metrics, it is crucial to both promptly identify faults and localize the root-cause service component. This is because rapid fault localization is critical to containment, stopping localized issues from propagating into cluster-wide cascade failures [9].

Although the academic community has proposed various methods for root cause analysis of software service anomalies, Edge-deployed distributed service systems face three distinct problems due to the dynamic and complex nature of their IoT application environments.

1. In edge networks, dynamic links and resource constraints induce random delays, jitter, and packet loss between sensors and edge datacenter. This non-deterministic interference causes multi-source data from the same event to arrive asynchronously. Consequently, these uncertain transmission characteristics impose nonlinear time shifts across data streams. These shifts fundamentally undermine the assumption of temporal alignment. This may lead to significant errors into feature extraction methods that require precise temporal alignment.
2. A fundamental issue in edge networks is the lack of a unified topological view. When a fault occurs, the dynamic dependencies among edge service instances cause rapid propagation along cascading fault chains. This propagation is further exacerbated by network retry operations, which cause non-linear amplification of the fault signals along its path.
3. A critical problem in edge environments is capturing low-frequency and high-risk faults in real time. This difficulty originates in constraint of limited computing resources. It is further compounded by a general absence of labeled historical fault data.

To rapidly localize the root cause of data anomalies, we propose ST-GraphRCA—a spatio-temporal graph propagation framework. It analyzes dependencies in an unsupervised environment to identify the anomalous service in edge networks. The main contributions of this work are as follows:

1. To address time-series misalignment in multi-source sampling metrics caused by edge network uncertainty, a novel PCA-DTW hybrid feature extraction method is proposed. Without requiring prior time-series synchronization, to improve multi-source feature extraction accuracy, this method addresses random transmission delays and stretching deformations via nonlinear vector structure alignment. The procedure is two-fold: First, metrics are categorized into network (throughput, RTT, retransmission rate) and computation (CPU utilization, memory usage, deadlock count, I/O) group. Second, Principal Component Analysis (PCA) is utilized to diagnose the primary cause of misalignment network or computation delays. This diagnosis then guides the Dynamic Time Warping (DTW) alignment strategy: a backward shift (insertion) is applied when network factors dominate, while a forward shift (deletion/compression) is used when computational delays prevail.
2. In edge environments, cascading faults can be rapidly amplified. To localize their root cause, we design a stream-based forward propagation graph based on the flow conservation principle. This graph models fault propagation through dynamic directed edges, which establish the primary anomaly path. Edge weights are dynamically adjusted. This suppresses interference from non-critical calls, such as heartbeats. Concurrently, the input and output anomaly quantities at each graph node are monitored to pinpoint the root cause source.
3. To detect low-frequency and high-risk anomaly faults in real time, we design a topology-constrained high-utility mining algorithm. A reachability pruning mask is first constructed from the forward propagation graph topology. The mask function is to focus high-utility mining on topologically feasible pathways. It achieves this by forcibly eliminating topologically unreachable candidate patterns during their generation. Furthermore, the utility function is optimized to enhance causal filtering capability of the algorithm. The key step is to suppress the utility values of passive victim nodes. These nodes exhibit high anomaly values alongside zero net outflow. This approach ensures the algorithm more accurately identifies the true root causes of low-frequency and high-risk anomaly faults.

## 2. Related Work

Research on root cause analysis for complex distributed IoT systems falls into three categories. These are causal inference-based methods, Large Language Model (LLM)-based methods, and deep learning-based methods.

1. The principle of causal inference is to construct causal graphs from observational data to achieve root cause analysis of anomaly sources. Pham utilized the classic Peter-Clark (PC) causal discovery algorithm to construct static causal dependencies from observational data, evaluating and comparing 9 causal discovery methods and 21 root cause analysis methods. Experimental results show that no single causal inference method is universally applicable. Thus, we need to design algorithms with strong adaptability. They should work for different application scenarios [10]. The existing LiNGAM algorithm fails to utilize sparse structures and high-order moment information. Harada proposed a new method to address this limitation. This method relies on a single statistical criterion. It improves the ICA log-likelihood and sparse penalty terms. However, edge networks face data transmission latency and jitter. It is difficult to satisfy the strict synchronization assumptions of LiNGAM [11]. To address the problem of lacking causal interpretability in IoT anomaly detection, Gad combined the LiNGAM causal discovery algorithm with interpretable Random Forest to identify causal relationships within network traffic data, thereby enhancing model interpretability in IoT scenarios. However, this method relies on extensive labeled data, making it difficult to adapt to complex and variable edge computing environments [12]. To address the problem of lacking causal interpretability in IoT anomaly detection, Gad combined the LiNGAM causal discovery algorithm with interpretable Random Forest. This identifies causal relationships within network traffic data. Thus, it enhances model interpretability in IoT scenarios. However, this method relies on extensive labeled data. Therefore, it is difficult to adapt to complex and variable edge computing environments [13]. Ikram proposed hierarchical and local learning methods to reduce the computational complexity of root cause analysis. These methods learn only the relevant parts of the causal graph. Thus, they reduce conditional independence tests. However, cascading fault chains form easily in edge networks. Therefore, local learning may overlook global propagation paths [14]. To rapidly identify root cause metrics, Li proposed a method that transforms analysis into an intervention recognition task. It first constructs a causal Bayesian network using system architecture knowledge. Then it monitors changes in the probability distributions of variables conditioned on their parents. This establishes causal relationships. However, the method relies on a pre-defined system architecture. Thus, it is hard to adapt to dynamic changes in edge networks [15]. For variable-level root cause analysis of anomalies, Budhathoki proposed a method based on causal graphs and functional causal models. This method utilizes counterfactual Shapley values to quantify each node’s contribution to an anomaly, in order to achieve attribution at the variable level. However, dependency associations among edge service instances are typically dynamic. This makes it difficult to define an accurate global causal graph in advance [16]. Orchard proposed a Polytree algorithm to address small-sample root cause analysis and the lack of structural knowledge. This method uses edge anomaly scores to perform root cause traversal. However, it relies on simple structural assumptions. Thus, it cannot address complex cascading fault chains [17]. In summary, while causal inference methods improve the interpretability of anomaly root cause analysis, they face notable challenges when applied to edge computing environments. First, uncertain edge network transmission induces jitter, which in turn creates nonlinear time shifts in asynchronously collected data. Second, the pervasive dynamic dependencies among microservices further complicate the establishment of stable causal models. The resulting temporal misalignment makes traditional correlation calculations ineffective. Consequently, critical anomaly features can be missed.
2. Root cause analysis based on Large Language Models (LLMs) uses semantic understanding to find root causes. Traditional methods are not only reliant on manual expertise but are also prone to incomplete variable sets and flawed causal assumptions. Tang introduced LLMs to address this problem. He used them to parse system logs and construct dynamic causal graphs. The LLM captures causal relationships and dynamic features across temporal dimensions. This achieves anomaly root cause localization [18]. Fine-tuning large language models (LLMs) for root cause analysis is costly and resource-intensive. To address this, Zhang proposed a method using GPT-4 and in-context learning. This method retrieves historical similar incidents and uses them as prompts to guide the model in localizing fault root causes. However, the method relies on the cloud-based GPT-4 model. Therefore, it cannot meet the real-time and data localization requirements of edge computing [19]. Fault analysis requires the generation of high-quality decision sequences. To achieve this, Ezukwoke addressed the fault analysis triplet generation task. The approach used a fine-tuned BERT-GPT2 model, which improved the coherence of the generated sequences [20]. Li proposed the COCA model to address incomplete fault reports submitted by users. It extracts diagnostic clues from code to reconstruct execution paths. This assists the LLM in root cause summarization and localization. However, this method lacks labeled historical data. Therefore, it is difficult to capture low-frequency, high-risk fault events in real-time [21]. Szandała evaluated the capability of Large Language Models (LLMs) to diagnose system faults. This evaluation used observational metrics within a chaos engineering framework. Benchmarking models like GPT and Gemini on fault diagnosis tasks showed that few-shot prompting can improve accuracy. However, LLMs have an inherent hallucination problem. This makes them unsuitable for highly reliable, real-time edge systems [22]. Goel proposed the eARCO framework to address the problem of static prompts in LLM root cause analysis. It automatically optimizes prompts and combines them with domain-adaptive Small Language Models (SLMs). However, SLMs also face computational bottlenecks on resource-constrained edge nodes [23]. There is a lack of benchmark datasets to evaluate the ability of LLMs to localize software fault root causes. Xu constructed the OpenRCA benchmark dataset. It contains 335 fault cases and massive telemetry data. However, the result shows that the currently best-performing large models have a low-resolution rate for complex faults, reaching only 11% [24]. Roy proposed the ReAct method for RCA agents. It addresses the inability to dynamically collect diagnostic information. This includes logs, metrics, and databases. However, using event report data as additional input did not yield significant performance gains [25]. Static feature extraction methods struggle to capture dynamic root cause patterns in event sequences. Zhu introduced TraceLM. This method uses context-embedded language models to directly capture temporal dynamic features from data. These features are then compared to localize the root cause. However, in edge environments, the method encounters a problem: non-linear time shifts in the sampled data [26]. Distributed Kubernetes containers face high complexity due to state consistency maintenance. Xiang proposed the SynergyRCA framework to resolve this problem. It constructs state graphs to capture spatio-temporal dependencies. When a fault occurs, the LLM combines expert prompts with dynamic graphs. It predicts the fault root cause. However, edge networks lack a global unified dynamic topology view. Thus, the state graph capture method has limitations [27]. In summary, LLM-based methods offer powerful semantic understanding for root cause analysis. However, their large model size and high inference latency make them difficult to deploy in edge network scenarios.
3. Deep learning-based methods use dependency graphs to extract topological features. They capture complex invocation relationships among software components. Qia addressed open graph anomaly detection by summarizing a set of widely used datasets [28]. Alsalman proposed the FusionNet model to improve IoT anomaly detection. This model combines Random Forest (RF), K-Nearest Neighbors (KNN), Support Vector Machine (SVM), and Multi-Layer Perceptron (MLP). However, integrating multiple models increases inference latency and resource consumption at the edge. Therefore, it is difficult to meet real-time requirements [29]. Lin proposed the RUN model to incorporate temporal features into root cause analysis. This model uses a contrastive learning encoder to capture complex dependencies among microservices in cloud data centers. However, it ignores anomaly propagation between nodes, which can lead to cascading issues [30]. Separately, Deng addressed the challenge of capturing high-dimensional temporal features. The method combines structural learning with Graph Neural Networks (GNNs) and uses attention mechanisms. This improves the interpretability of anomaly detection, helping users infer root causes. A key limitation is that the method cannot capture low-frequency, high-risk fault events [31]. Steenwinckel proposed the FLAGS method to fuse data-driven and knowledge-driven root cause analysis. It integrates semantic knowledge based on machine learning and utilizes user feedback for adaptive optimization. Thus, it improves the interpretability of root cause localization and reduces knowledge graph modeling costs [32]. Klein utilized knowledge graphs to derive component dependencies in distributed software. He input these into a Siamese Graph Convolutional Network (GCN). This model diagnoses anomalies and achieves localization via graph pattern matching. However, static knowledge graphs struggle to adapt to dynamic topology structures in edge networks [33]. Nadim discovered state-event graphs from low-level continuous observational data, extracting highly accurate and trustworthy patterns from raw data, and finally generated causal models from event logs [34]. Nadim discovered state-event graphs from low-level continuous observational data. This approach extracts reliable patterns from raw data and generates causal models from event logs [35]. Separately, defining rules for Complex Event Processing (CEP) in IoT is challenging. Simsek proposed an automated framework to address this. The framework uses deep learning for rule extraction and includes data labeling and rule extraction components [36]. The root of misjudgment lies in the fact that passive nodes may exhibit higher anomaly metrics than the true fault source—a scenario where single-feature analysis inherently fails to identify the correct origin.

In summary, root cause analysis for anomalies in resource-constrained edge IoT environments faces three unsolved problems:

1. Time-Series misalignment from physical factors. Existing methods often assume multi-sensor data is time-aligned or lack proper alignment strategies for different data types [11,12]. However, in edge systems, time shifts occur due to specific physical reasons. Not accounting for this during data alignment causes distortion. This breaks the true relationships between features and leads to missing important anomaly evidence.
2. Uncaptured dynamics in cascading faults. For cascading faults, traditional dependency graphs map node connections but ignore the direction and strength of fault spread [30]. Passively affected nodes can show higher anomaly scores than the source node. This makes it difficult to identify the true root cause—characterized by high net fault output—using simple feature comparisons.
3. Search space explosion and causal confusion. High-Utility Mining (HUM) is used on the edge to find rare, high-risk faults [37]. Standard HUM algorithms check all possible combinations without limits, causing an exponential growth in search space that overwhelms edge devices. Furthermore, standard utility measures cannot filter by cause. They give high scores to severely affected victim nodes, causing both missed real faults and false alarms for high-risk patterns.

## 3. Model Methodology

### 3.1. Model Structure

**Figure 1:**
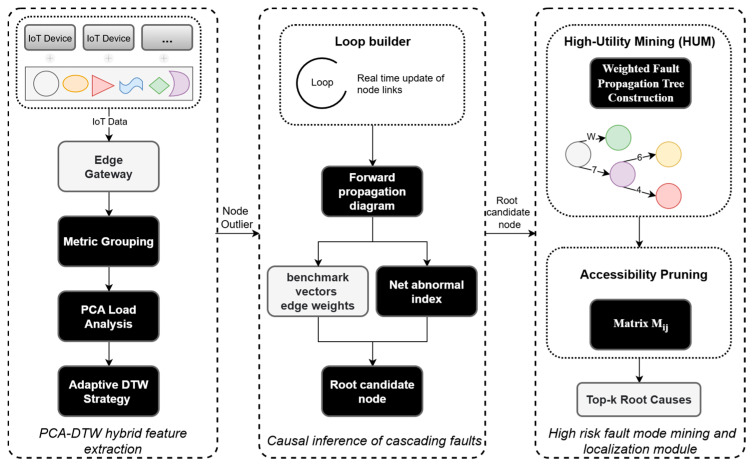
System Framework Diagram.

The structure of the ST-GraphRCA model comprises three modules: PCA-DTW hybrid feature extraction, cascading fault causal inference, and anomaly fault analysis and localization, as illustrated in Figure 1.

PCA-DTW Hybrid Feature Extraction

The feature extraction process is as follows:

1. Various system operation and maintenance data transmitted via the edge network are received and aggregated to form a multi-dimensional metric matrix.
2. The metric data are grouped into network and computation categories. Subsequently, environmental noise is filtered and principal components are extracted using an online PCA method [38].
3. Based on the characteristics of the different groups, the DTW temporal alignment strategy is dynamically adjusted to achieve elastic alignment between observation sequences and reference vectors.
4. Based on the DTW calculation, the minimum warping value at the end point of the cumulative cost matrix is taken as the node’s anomaly score, which completes the node feature extraction. By addressing the temporal misalignment caused by non-deterministic edge interference, this module significantly improves feature extraction accuracy.

Cascading Fault Causal Inference

The causal inference process for the direction of cascading fault propagation in distributed service environments is as follows:

1. A forward propagation graph is constructed. Node anomaly scores are obtained from the feature extraction module, and the weights of service invocation edges are updated by combining streaming trace data. The model maintains the directed edges of the forward propagation graph in real time to determine the propagation path of anomaly faults.
2. The reference vectors are maintained based on the node anomaly scores extracted via PCA-DTW. The anomaly score serves as a real-time metric for quantifying the node anomaly status.
3. For each node in the forward propagation graph, the net inflow energy is calculated using the weights of inflow edges and the corresponding node anomaly scores, while the net outflow energy is calculated using the weights of outflow edges and the corresponding node anomaly scores. Finally, the net anomaly outflow metric is calculated by $\Sigma_{out}-\Sigma_{in}$. This net anomaly outflow metric is utilized to infer the root cause candidate node of a single propagating anomaly fault chain. As shown in Figure 1, The model is grounded in the principle of flow conservation. This principle allows it to quantify fault propagation intensity. Based on this quantification, it pinpoints active fault sources by their positive net outflow and differentiates them from passive victims, which exhibit negative or zero outflow.

Anomaly Fault Analysis and Localization

A topology-constrained high-utility mining algorithm is designed to complete anomaly root cause localization. The specific process is as follows:

1. A reachability constraint matrix is first constructed from the topological connections of the forward propagation graph. This matrix then provides a definitive strategy for pruning unreachable paths during the mining process.
2. To assess the likelihood of a root cause, we define a utility function for candidate nodes that show net outflow anomalies. This function is intentionally designed with two complementary parts. The first part is the internal utility, which is based on the inflow edge weights and the node’s anomaly score. The second part is the external utility, based on the outflow edge weights and the anomaly score. This two-part design allows for a detailed evaluation of fault influence in both the incoming and outgoing directions. The total utility, calculated as the sum of these two components, provides a single, consolidated measure for root cause identification.
3. The process generates an initial set of candidate patterns. These patterns are then filtered using a constraint matrix. The purpose of this matrix is to eliminate any combinations that are topologically unreachable. This step ensures computational efficiency and helps prioritize the detection of low-frequency, high-risk anomaly sources. After this filtering, a recursive high-utility pattern mining algorithm operates within the constrained solution space. This algorithm performs real-time utility calculations for each candidate path. As a result, the final output is refined to include only the high-utility patterns that also possess the critical signature of high net outflow, which is indicative of a root cause.
4. The screened high-utility anomaly node sequence is output, completing the root cause localization.

### 3.2. PCA-DTW Hybrid Feature Extraction

The objective of this section is to address the extraction of anomaly features from multi-source operation and maintenance (O&M) data. In edge networks, transmission interference is inherently stochastic. This interference causes nonlinear time shifts in data aggregated from multiple sources. As a result, the effectiveness of feature extraction methods is fundamentally undermined, as these methods rely on strict temporal alignment to work properly. This constitutes the key problem to be resolved in this section.

**Figure 2:**
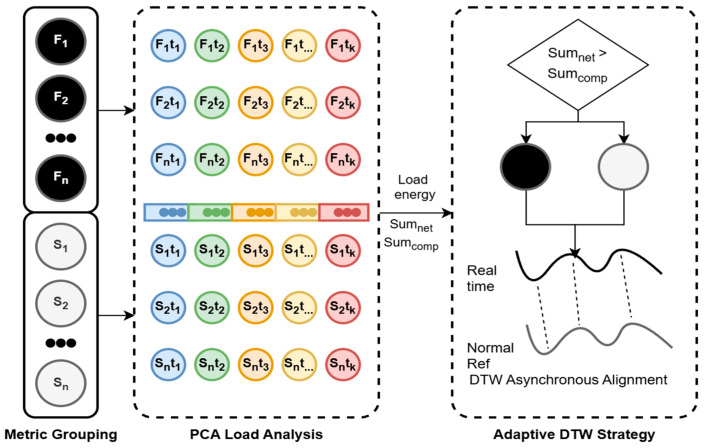
PCA-DTW hybrid feature extraction diagram. (Dashed lines denote the aggregation of multiple functional modules, whereas solid lines represent individual specific modules.)

The structure of the PCA-DTW feature extraction module is illustrated in Figure 2. The primary working mechanisms of this model are described as follows:

1. Physical Semantic Grouping: Grouping is performed at the input stage based on the distinct physical semantics of the operation and maintenance data. As shown on the left side of Figure 2, the raw high-dimensional collected data are mapped into two semantic subspaces: the network-sensitive group $G_{net}$ (containing $F_{1}\ldots F_{n}$, colored black, such as network throughput, RTT, retransmission rate, etc.) and the computation-sensitive group $G_{comp}$ (containing $S_{1}\ldots S_{n}$, colored white, such as CPU utilization, memory usage, deadlock count, etc.
2. Energy Attribution Diagnosis: Firstly, within the $t_{1}\ldots t_{k}$ time period, data from the network group (black F) and the computation group (white S) are projected into a low-dimensional space to extract the first principal component. The sum of the absolute values of the loading energies for each metric within the first principal component vector is calculated. Secondly, the loading energy is calculated by group. Specifically, for the network group energy $E_{net}$ (labeled as Sum_net_ in the figure), the sum of the weights of all network metrics ($F_{1}t_{1}\ldots F_{N}t_{k}$) within the principal component is calculated; for the computation group energy $E_{comp}$ (labeled as Sum_comp_ in the figure), the sum of the weights of all computation metrics ($S_{1}t_{1}\ldots S_{N}t_{k}$) within the principal component is calculated.
3. Gated DTW Alignment Algorithm: As illustrated on the right side of Figure 2, upon acquiring the loading energies, the process enters the decision loop depicted on the right. By comparing the loading energy proportions between the network and computation groups, we determine the physical root cause of the fault in real time for the current window. The diagnostic result serves as a control signal for the DTW algorithm module. When the fault is determined to be network-dominated, the left branch is activated, which reduces the DTW insertion penalty. This adjustment allows the algorithm to adapt to data lag (e.g., from network congestion) by applying a backward shift. Conversely, When the fault is determined to be computation-dominated, the right branch is activated, reducing the DTW deletion penalty. This enables the algorithm to handle data loss (e.g., from CPU deadlocks) via forward compression. Finally, the anomaly score is calculated by aligning the real-time vector with the reference vector.

**Figure 3:**
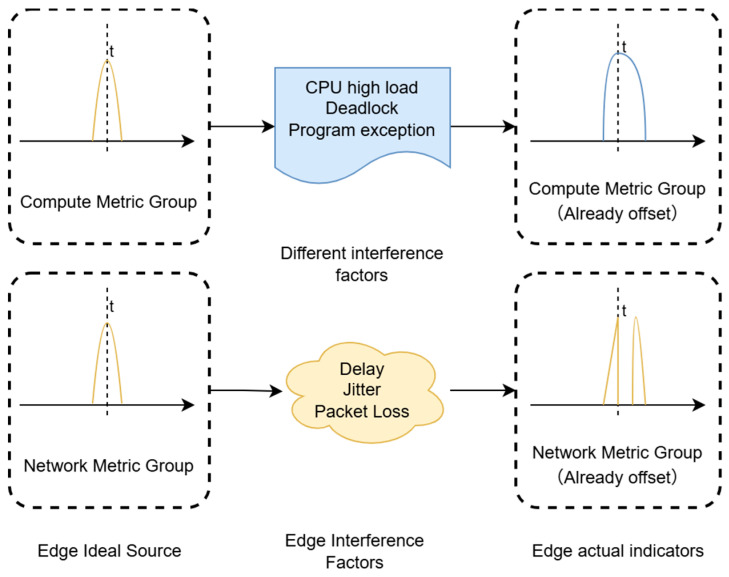
Schematic diagram of heterogeneous data temporal misalignment. (Dashed lines represent the aggregation of metric groups, and solid lines denote individual functional components. Arrows indicate the process flow and the impact of interference factors. Different colored lines represent distinct metric categories (e.g., blue for compute metrics and yellow for network metrics), and t denotes the time dimension.)

Within the feature extraction module, a critical problem exists: the temporal misalignment encountered during the aggregation of distributed multi-source operation and maintenance (O&M) data. As illustrated in Figure 3, when a system fault occurs, network-type metrics at the source (such as network throughput, RTT, and retransmission rate) and computation-type metrics (such as CPU utilization, memory usage, deadlock count, and I/O) are triggered synchronously in physical time (labeled as “Edge Ideal Source” in Figure 3). However, following transmission through unstable edge networks or due to computational faults at edge nodes, the metric data collected by the gateway exhibit distinctly different distortion characteristics. (1) Network Group Shift. Network metrics are typically influenced by link jitter, manifesting as waveform stretching and overall lag; such as network jitter occurring at time *t* result in waveform fractures or data absence at point *t*. (2) Computation Group Shift. Computation metrics are more heavily influenced by processing blocking or packet loss, manifesting as missing data points or non-linear local delays; for example, factors occurring at time *t* lead to waveform amplification at point *t*. To address this problem, the PCA-DTW feature extraction method is designed. The specific details of this method are as follows:

1. From a spatial perspective, the metrics for anomaly judgment are grouped according to their distinct physical meanings. Let the standardized input matrix be denoted as $X\in R^{N\times T}$, containing the multi-dimensional sensor time-series data matrix (including CPU, memory, RTT, etc.). N represents the system metric dimension aggregated from edge nodes, and T (Initialize to 60 s) denotes the time step length of the sliding window. Based on physical characteristics, the data are divided into the network group $G_{net}$ and the computation group $G_{comp}$.
2. PCA is adopted for dimensionality reduction to improve the computational efficiency of the algorithm and obtain the loading energies of each group. First, the covariance matrix Σ is calculated:
  (1) $$\Sigma =\frac{1}{T-1}XX^{T}$$

By performing eigenvalue decomposition on, the eigenvector of the current first principal component $v_{1}$ is obtained, along with the eigenvector principal component matrix $W_{k}\in R^{N\times k}$ corresponding to the largest k (Take k = 1) eigenvalues. Statistical signal processing theory gives us guidance. In high-dimensional sensor data streams, the first principal component usually explains most of the variance (over 85%). So it captures the main anomaly trend of the system. Higher-order components often contain random environmental noise [39,40]. Edge nodes have limited computing resources. To best balance detection accuracy and inference latency, this work uses the first principal component as the feature vector $v_{1}$. We further tested this choice in the parameter sensitivity experiments in Section 4.5. The results confirm it is a reasonable choice. Next, utilizing the eigenvector of the first principal component at the current timestamp $v_{1}$, the contribution proportions of the two groups of metrics are calculated: the network loading energy $E_{net}=\sum_{i\in G_{net}}|v_{1}^{\left(i\right)}|$ is defined as the sum of the projection energies of the network group $G_{net}$ for all network metrics on the current principal component; the computation loading energy $E_{comp}=\sum_{j\in G_{comp}}|v_{1}^{\left(j\right)}|$ is defined as the sum of the projection energies of the computation group $G_{comp}$ for all computation metrics on the current principal component.

3. From a temporal perspective, a gated DTW alignment method is designed to calculate node anomaly scores. Let the real-time feature sequence obtained within time T be denoted as $Q=\{q_{1},q_{2},\ldots ,q_{T}\}$, and the historically maintained forward reference sequence be denoted as $R=\{r_{1},r_{2},\ldots ,r_{T}\}$ (where $q_{i},r_{j}\in R^{k}$). First, a local distance matrix D of size T×T is constructed, where the element D(i,j) represents the distance between two points:
  (2) $$D(i,j)=\|q_{i}-r_{j}{\|}_{2}$$

To find the optimal alignment path between two sequences, the minimum cumulative distance matrix C is calculated. Its recursive formula is defined as follows:

(3) $$C(i,j)=D(i,j)+\min\left\{\begin{array}{ll} \alpha \cdot C(i-1,j-1) & \left(\mathrm{Match}:\;\mathrm{Sync}\right)\\ \beta \cdot C(i,j-1) & \left(\mathrm{Deletion}:\;\mathrm{Skip}/\mathrm{Loss}\right)\\ \gamma \cdot C(i-1,j) & \left(\mathrm{Insertion}:\;\mathrm{Lag}/\mathrm{Latency}\right) \end{array}\right.$$

Here, let α,β,γ be the DTW directional penalty factor. According to the PCA loading energy analysis results, if $E_{net}>E_{comp}$ (typically manifesting as data delayed arrival), the value of $\gamma \leftarrow \gamma_{low}$ is reduced; if $E_{net}<E_{comp}$ (typically manifesting as data loss), the value of $\beta \leftarrow \beta_{low}$ is reduced. In the standard state, α=β=γ=1 represents standard DTW, The initial value for adjusting the penalty factor change rate is set to $△\gamma_{low}=0.45$, $△\beta_{low}=0.57$; when $E_{net}>E_{comp}$ indicates network dominance, γ<1 is set to reduce the lag penalty to adapt to transmission latency; when $E_{comp}>E_{net}$ indicates computation dominance, β<1 is set to reduce the skip penalty to smooth sampling loss. The three options in this recursive formula correspond respectively to three topological transformation operations of the time series during the alignment process, the meanings of which are analyzed as follows:

1. Match: Corresponds to diagonal movement. This implies that time step i of the real-time sequence is perfectly aligned with time step j of the reference sequence. A smoothly operating edge node is indicated by three key conditions: minimal disparity in PCA loading energy between network and computation groups, no significant transmission jitter, and no sampling blockage. Together, these ensure strict synchronization between sensor data generation and processing.
2. Compression: Corresponds to horizontal movement. This operation is activated by the computation group, implying that the real-time sequence skips certain segments of the reference sequence. This corresponds to scenarios of sampling loss or blockage, when edge devices fail to generate partial data points due to CPU deadlocks or high load, reducing the penalty allows DTW to automatically eliminate the corresponding missing segments in the reference, achieving elastic compression alignment of discontinuous data.
3. Insertion: Corresponds to vertical movement. This operation is activated by the network group, implying that a single point in the real-time sequence maps to multiple points in the reference sequence, forming a stretching effect. By reducing the penalty, the system actively identifies this temporal distortion caused by environmental lag to distinguish it from genuine numerical anomalies and prevent misdiagnosis.

Finally, upon completion of the DTW topological transformation operations, the cumulative matrix C completes the traversal of the entire temporal space. The element at the terminal of the diagonal represents the anomaly score between the real-time sequence Q and the reference sequence R under the optimal topological transformation path; this constitutes the final feature extracted by the PCA-DTW algorithm for a single node. In subsequent modules, this value is utilized as the initial anomaly score of the node and input into the stream-based forward propagation graph. The PCA-DTW hybrid feature extraction method is presented in Algorithm 1.**Algorithm 1.** PCA-DTW hybrid feature extraction.**Require**: $X\in R^{N\times T},\;R=\{r_{1},\ldots ,r_{T}\},\;G_{net},\;G_{comp}$ Compute Covariance Matrix: $\Sigma \leftarrow \frac{1}{T-1}XX^{T}$ Perform Eigendecomposition: ΣV extract $v_{1}$ Generate Projected Sequence: $W_{k}\in R^{N\times k}$ Calculate Semantic Energy Contributions: $E_{net}\leftarrow \sum_{i\in G_{net}}|v_{1}^{\left(i\right)}|,\;E_{comp}\leftarrow \sum_{j\in G_{comp}}|v_{1}^{\left(j\right)}|$ Initialize Penalty Factors: α←1,β←1,γ←1 **If** $E_{net}>E_{comp}$ **then** $\gamma \leftarrow \gamma_{low}$ **else if** $E_{comp}>E_{net}$ **then** $\beta \leftarrow \beta_{low}$
Initialize Cumulative Matrix $C\in R^{T\times T}$ **for** i = 1 to T **do** **for** j = 1 to T **do** $Cost_{match}\leftarrow \alpha \cdot C(i-1,j-1)$ $Cost_{skip}\leftarrow \beta \cdot C(i,j-1)$ $Cost_{lag}\leftarrow \gamma \cdot C(i-1,j)$ $C(i,j)\leftarrow ||q_{i}-r_{j}|{|}_{2}+\min(Cost_{match},Cost_{skip},Cost_{lag})$      **return** Normalized Score S(v) based on C(T,T)


ST-GraphRCA uses a dynamic programming mechanism to identify a minimum warping path with low time complexity. This path is then used to quantify the similarity between the real-time state and the normal state within the non-linear temporal dimension. This effectively mitigates the sensitivity of vector distance to temporal shifts.

### 3.3. Cascading Fault Causal Inference

**Figure 4:**
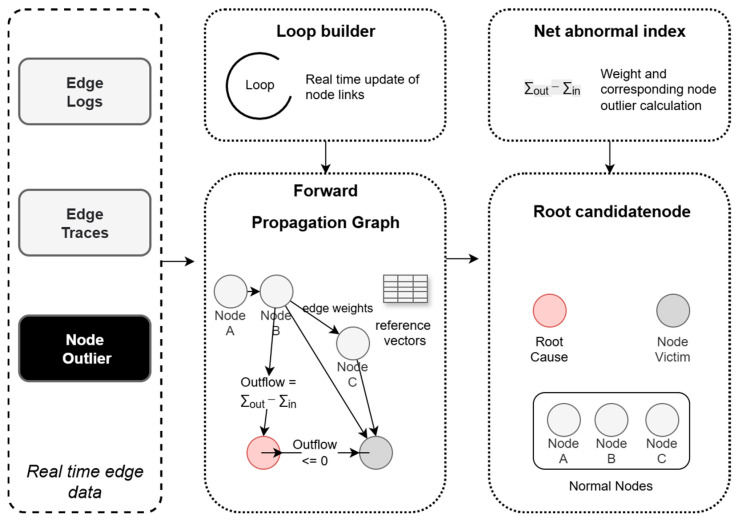
Causal inference of cascading faults Diagram.

The objective of this section is to infer candidate solutions for the root causes of anomaly faults within cascading fault chains. A cascading fault causal inference method based on the principle of flow conservation is designed. The specific working principle of this method is as follows:

1. Real-time Data Aggregation. As illustrated on the left side of Figure 4, real-time data streams containing invocation traces (Traces) and logs (Logs) are received from the edge. This data is then used to parse the dynamic invocation relationships among microservices. Simultaneously, anomaly scores output by the PCA-DTW hybrid feature extraction module are accepted; these scores quantify the current degree of anomaly for individual nodes.
2. Construction of Dynamic Forward Propagation Graph. As illustrated in the center of Figure 4, the connection relationships between nodes are updated in real time utilizing streaming data, and node states are defined based on anomaly scores. The graph structure is dynamically updated from the trace stream. A new edge is created when a new invocation appears. Conversely, if an invocation is absent for an extended period, the corresponding edge is removed via a weight decay mechanism. Under normal conditions, the system maintains two key elements in real time. First, it updates node reference vectors based on the latest vector data. Second, it adjusts node edge weights based on the ongoing Trace stream activity.
3. Causal Inference of Root Cause Candidate Nodes. As illustrated on the right side of Figure 4, the net inflow and outflow anomaly indices of nodes are first calculated based on the anomaly scores and edge weights within the forward propagation graph. If the net outflow of a node exceeds a safety threshold τ, it indicates that not only is the anomaly severity of the node itself high, but the anomaly energy propagated outward is also significantly greater than that received; consequently, it is determined to be an active fault source, as indicated by the red node in the figure. Conversely, this implies the node is merely affected by upstream faults; therefore, it is determined to be a passive victim and is eliminated, as indicated by the gray node in the figure. Finally, the anomaly candidate nodes are output.

The specific content of the causal inference module is detailed below. The first problem is how to construct and maintain the dynamic forward propagation graph. The forward propagation graph is defined as a directed graph $G_{t}=(V_{t},E_{t},W_{t})$, wherein:

1. $V_{t}$ (Node Set): Represents the edge services or components active within time window *t*. Each node $v_{i}\in V_{t}$ in the graph stores a reference vector R. This state vector corresponds to the normal reference vector extracted via DTW-PCA for the network and computation groups under normal conditions, as described in Section 3.2; it represents the feature set of the node during normal operation. The update algorithm is designed with a dual goal: it must adapt to inherent edge variations like workload fluctuations and hardware aging, without allowing sudden fault data to corrupt the reference baseline. Assume that at time step *t*, the real-time observation vector of node $v_{i}$ after PCA dimensionality reduction is $x_{t}^{\left(i\right)}$. $R_{t-1}$ denotes the set of reference vectors prior to time *t* − 1. For any node $v_{i}$ in the forward propagation graph, its confidence $S_{t}$ is defined as:
  (4) $$S_{t}^{\left(i\right)}=Sigmoid\left(\frac{C_{final}(v_{i})-\mu_{history}}{\sigma_{history}}\right)$$
  where $C_{final}(v_{i})$ is the node anomaly score of node $v_{i}$ (i.e., the terminal value $C_{final}(v_{i})=C(T,T)$ of matrix C in Section 3.2), and $\mu_{history}$ and $\sigma_{history}$ are the rolling mean and standard deviation of historical anomaly scores, initialized to the values upon first entry. The Sigmoid function maps the score to the (0,1) interval, representing the confidence that the node is in an anomalous state. The reference is allowed to update only when the data confidence is below the safety threshold τ (set to 0.8); otherwise, the reference update is frozen. During the update, a smoothing factor ψ (set to 0.02) is introduced, and an exponential moving average algorithm is employed to reduce the impact of low-frequency sporadic connections. This enables the model to track normal environmental drift while maintaining robustness against contamination from sudden fault data. The reference vector update algorithm is presented in Algorithm 2.
**Algorithm 2.** Stream-based Graph Reference Vector Maintenance.**Require**: $X_{t}=\{x_{t}^{\left(1\right)},\ldots ,x_{t}^{\left(k\right)}\}$ Previous reference vectors $R_{t-1}=\{r_{t-1}^{\left(1\right)},\ldots ,r_{t-1}^{\left(k\right)}\}$ Anomaly Threshold τ, Smoothing Factor ψ Anomaly Scores $S_{t}$ **for** each node $v_{i}$ in active microservices **do** $S_{t}\leftarrow C_{final}(v_{i})$
**if** $S_{t}<\tau$ **then** $r_{t}^{\left(i\right)}\leftarrow (1-\psi )\cdot r_{t-1}^{\left(i\right)}+\psi \cdot x_{t}^{\left(i\right)}$ **else** $r_{t}^{\left(i\right)}\leftarrow r_{t-1}^{\left(i\right)}$ **return**
$R_{t},S_{t}$
2. $E_{t}$ (Edge Set): Represents the forward invocation dependency relationships among services. A directed edge $e_{ij}=(v_{i},v_{j})\in E_{t}$ indicates that service $v_{i}$ initiated a request that propagated to $v_{j}$. These edges are dynamically updated by parsing the relationship between the parent node ID and the node ID within the Trace stream in real time.
3. $W_{t}$ (Weight Set): The edge weight $w_{ij}\in [0,1]$ represents the confidence intensity of the dependency relationship. During the continuous influx of trace streams, certain service invocations may be sporadic (such as heartbeat detection or one-off tasks); however, faults typically propagate along high-frequency dependency paths. Therefore, dynamic weighted updating based on streaming data is required. To quantify the gradation in dependency, the system employs an exponential moving average (EMA) algorithm for real-time edge weight updates. When a new trace data stream arrives and an invocation of $v_{i}\to v_{j}$ is detected, the weight update formula is:
  (5) $$w_{ij}^{\left(t\right)}=\psi \cdot 1+(1-\psi )\cdot w_{ij}^{\left(t-1\right)}$$

If no invocation is detected within the time window Δt, the weight decays naturally:

(6) $$w_{ij}^{\left(t\right)}=(1-\psi )\cdot w_{ij}^{\left(t-1\right)}$$

where ψ (set to 0.02) is the smoothing factor. This mechanism enables the propagation graph to respond rapidly to new service traffic patterns while suppressing low-frequency sporadic noise interference. For example, when service migration or scaling makes an old dependency invalid, the model quickly reduces the weight of the corresponding edge to zero. This removes the old topology. At the same time, the model uses Formula (5) to quickly capture any newly established call relationships.

The second problem is how to implement causal inference. The specific principle is as follows: Upon completion of the real-time update of the forward propagation graph, the net anomaly outflow metric ΔScore is calculated based on the principle of fault energy conservation. That is, for any node $v_{i}$ in the forward propagation graph $G_{t}$, the difference between its anomaly outflow $E_{out}(v_{i})$ and anomaly inflow $E_{in}(v_{i})$ is determined. A passive victim node in a fault chain exhibits a balance between the anomaly energy it receives and forwards. Conversely, an active fault source generates anomaly energy, resulting in an output that substantially exceeds its input. Therefore, the accumulated input anomaly energy and output anomaly energy for node $v_{i}$ are calculated respectively as follows:

(7) $$E_{in}(v_{i})=\sum_{v_{k}\in Parents(v_{i})}S(v_{i})\times w_{ij}^{\left(t\right)}$$

(8) $$E_{out}(v_{i})=\sum_{v_{j}\in Children(v_{i})}S(v_{j})\times w_{ij}^{\left(t\right)}$$

where S(v) is the anomaly score of the corresponding parent or child node, and $w_{ij}^{\left(t\right)}$ is the maintained dynamic edge weight. Net Anomaly Outflow Calculation: Finally, the net anomaly outflow $\Delta Score(v_{i})$ of node $v_{i}$ is defined as the difference between output and input:

(9) $$\Delta Score(v_{i})=E_{out}(v_{i})-E_{in}(v_{i})$$

Wherein, if $\Delta Score(v_{i})>\varphi$ (where φ is the set threshold, set to 0.42): this indicates that the anomaly diffused downstream by $v_{i}$ is far greater than that received from upstream, suggesting it may be an active fault source. Conversely, if the opposite is true, it indicates that $v_{i}$ is merely a medium for transmitting anomalies or a passive victim (likely a victim node); this serves as an important parameter for mining anomaly nodes in the root cause localization module.

**Figure 5:**
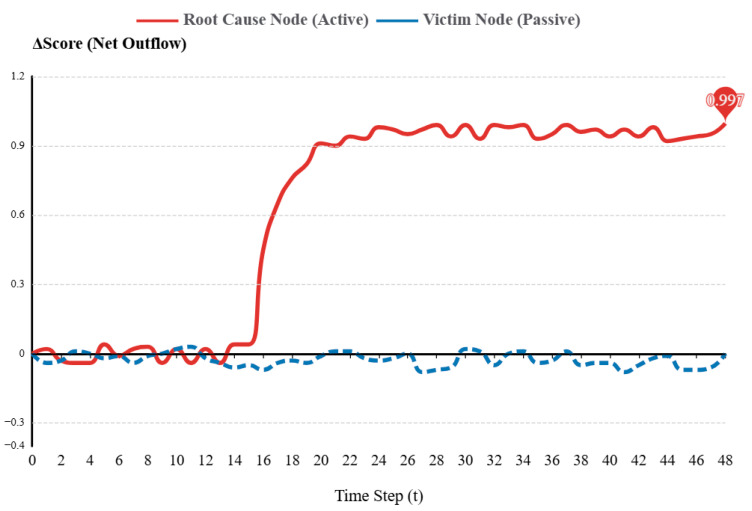
ΔScore dynamic evolution diagram.

Figure 5 shows the dynamic change in net anomalous outflow scores over time. This is for the root cause node and a downstream victim node during a single fault injection experiment. After the fault is injected at t = 15, the ΔScore of the root cause node quickly rises. It then stays in a high positive range (>0.8). This happens because the node is the fault source. It sends out a large amount of anomalous traffic but receives almost none. This makes its net outflow strongly positive. In contrast, the ΔScore of the victim node always fluctuates near 0 or shows a slightly negative value. This difference shows that ΔScore can effectively identify passive nodes and active fault sources in a cascading fault chain. Using this method, ST-GraphRCA can effectively control the size of the propagation graph. This ensures the real-time update of the forward propagation graph calculation. It also reduces the misjudgment rate caused by faults that amplify quickly along the cascade chain.

### 3.4. Anomaly Fault Analysis and Localization

The objective of this section is to achieve accurate localization of low-frequency, high-risk anomaly faults. Based on the streaming dynamic forward propagation graph $G_{t}=(V_{t},E_{t},W_{t})$, an anomaly fault localization method with topology reachability constraints is designed. This method comprises the following: (1) The causal utility function is defined on the forward propagation graph. It has two parts: the internal utility and the external utility. The internal utility for a node is calculated by multiplying the node anomaly score by the sum of its incoming edge weights. In contrast, the external utility is calculated by multiplying the same anomaly score by the sum of its outgoing edge weights. A node overall utility is the sum of its internal and external utilities. This overall utility is used to evaluate the node potential as a root cause. (2) Pruning Mask for Topology Reachability. A reachability constraint matrix is constructed based on the topological connection relationships of the forward propagation graph, providing a strategy for pruning operations to eliminate invalid non-causal combinations. (3) Root Cause Localization. To ensure efficiency, a constraint matrix first filters the candidate patterns. It does this by removing combinations that are topologically unreachable. This step prioritizes the search for low-frequency, high-risk root causes. Next, a recursive high-utility mining algorithm operates within this constrained solution space. The algorithm performs real-time utility calculations. It refines the pattern set to include only those patterns that have both high computed utility and strong net outflow characteristics. The final output is a screened sequence of high-utility anomaly nodes. This sequence precisely pinpoints the root cause, which concludes the localization process. The working principle of this method is detailed below:

1. Definition of Causal Utility Function

In fault localization scenarios, to suppress high-scoring victim nodes and highlight concealed root causes, the utility function is optimized utilizing the net anomaly outflow $\Delta Score(v_{i})$ calculated in Section 3.3. The utility of a node $v_{i}$ within a fault propagation chain is defined as comprising two components:

(10) $$eu(v_{i})=\mathrm{Max}(\alpha ,\Delta Score(v_{i}))$$

Here $eu(v_{i})$ represents the external utility, which is mapped to the net anomaly outflow of the node. ρ (set to 0.05) serves as a safety threshold to prevent excessively small utility values, thereby suppressing interference from non-causal noise. Max denotes the maximum value operation; if $\Delta Score(v_{i})\leq \rho$ indicates that the node is a victim or normal, its external utility is filtered out, effectively removing nodes that passively receive anomalies.

(11) $$iu(v_{i},T)=w_{parent(v_{i}),v_{i}}^{\left(t\right)}$$

Here, $iu(v_{i},T)$ represents the internal utility, which is mapped to the association confidence of the node within the current propagation path. Let T be the propagation chain; for a node $v_{i}$ within chain T, its internal utility corresponds to the edge weight $w_{parent(v_{i}),v_{i}}$ directed toward that node.

Consequently, for a propagating fault chain $X=\{v_{1},v_{2},\ldots ,v_{k}\}$, the total utility within trajectory T is defined as the weighted sum of all valid root cause nodes along the path:

(12) $$u(X,T)=\sum_{v_{i}\in X\wedge v_{i}\in T}eu(v_{i})\times iu(v_{i},T)$$

2. Design topology reachability pruning mask

This work addresses the complex search space problem of standard HUM algorithms in edge computing by designing a novel topology reachability pruning mask. A reachability pruning mask matrix M is constructed by utilizing the topological structure of the forward propagation graph $G_{t}$. Let *V* be the set of all services in the system, and let M be a Boolean matrix of size V×V. If and only if a directed path exists from node $v_{i}$ to $v_{j}$ in the forward propagation graph $G_{t}$, and the weight value $w_{ij}>0$ from node $v_{i}$ to $v_{j}$ satisfies the condition then $M_{ij}=1$ is set; otherwise, $M_{ij}=0$ is set. During the process of generating candidate patterns via the high-utility mining algorithm, for a candidate pattern $P=(v_{1},v_{2},\ldots ,v_{k})$ (initialized as empty) and a new node $v_{new}$ to be added, the generation of a new pattern $P_{new}=(v_{1},v_{2},\ldots ,v_{k},v_{new})$ is permitted only when the following topology reachability condition is satisfied:

(13) $$(\exists v_{i}\in P)M_{v_{i},v_{new}}=1$$

This constraint defines a topology reachability subspace, compelling the algorithm to perform depth-first search only on physically connected topological branches. For branches of $M_{ij}=0$, The computationally intensive high-utility weighted utilization step is circumvented, thus achieving exponential spatial pruning.

3. Localization of Anomaly Fault Root Causes

Input the stream forward propagation graph $G_{t}$. The utility upper bound TWU(v) of the node high-utility algorithm is defined as the sum of eu(v); utilizing a preset threshold δ (Initialize to 214), pre-pruning is implemented, and low-potential nodes TWU(v)<δ are eliminated from the global candidate set; to efficiently localize fault propagation chains during depth-first search, the TR-Mine algorithm is invoked to perform recursive expansion mining. The specific process is as follows:

- When attempting to append a new node $v_{new}$ to the terminal of the current fault chain P, the mask M is first queried. If $M_{last(P),v_{new}}=0$, indicating that the two nodes are unreachable on the forward graph, this invalid branch is pruned.
- For a new pattern $P_{new}$ that passes validation, if its utility upper bound TWU satisfies the threshold requirement, a projected database $D{|}_{P_{new}}$ is constructed, i.e., a sub-dataset containing only the subsequent propagation paths of $P_{new}$.
- $TR-Mine(P_{new},D{|}_{P_{new}})$ is invoked to enter the next level of recursion, continuing to search for deeper root cause nodes within the reduced subspace until no higher-value patterns are generated, outputting the Top-k candidate set nodes. Anomaly fault analysis and localization is presented in Algorithm 3:
**Algorithm 3.** Abnormal fault analysis and localization.**Require**: $G_{t}=(V_{t},E_{t},W_{t})$ **for** v∈V do eu(v)←max(α,ΔScore(v)) **do** eu(v)←max(α,ΔScore(v)) **end for** Construct Matrix M:$M_{ij}=1$ **if** i can reach j in $G_{t}$, **else** 0. Calculate TWU(v) using reshaped eu(v) $V_{valid}\leftarrow \{v|TWU(v)\geq \delta \}$ Sort $V_{valid}$ by TWU order Define TR−Mine: **for** node $v_{new}\in V_{valid}$ in P **do** **if** P≠ NULL and $M_{last(P),v_{new}}==0$ **then** New Pattern $P_{new}\leftarrow P\cup \{V_{new}\}$ **if**
$Utility(P_{new})\geq \delta$ **then** $P_{new}$ to $R_{final}$ **if**
$UpperBound(P_{new})\geq \delta$ **then** $TR-Mine(P_{new},D{|}_{P_{new}})$ **return** Top-k nodes in $R_{final}$ sorted by score descending

The algorithm is designed to efficiently mine anomaly root causes. It firstly performs an efficient search constrained to the plausible causal subspace. Then it outputs a focused Top-k set of high-utility candidates. This efficient approach is purposefully aligned with the limited resources of edge networks.

## 4. Experiments

### 4.1. Experimental Setup and Datasets

To validate the effectiveness of ST-GraphRCA in real-world IoT edge environments, an edge computing testbed based on Kubernetes was established. It simulates a high-concurrency backend for IoT data processing. The environment uses 32 physical nodes with components deployed in Docker containers. Kubernetes acts as the orchestration layer, managing a dynamic Apache Spark (v3.5.0) cluster as the edge computing nodes.

Then an OpenTelemetry-compatible monitoring scheme is implemented, which uses the ELK Stack and Beats. This setup collects cluster Logs, Traces, and Metrics (e.g., CPU, Memory, IO for hosts and containers) in real time.

The simulated load involves continuous edge IoT sensor streaming tasks. These tasks mimic the real-time cleaning, aggregation, and analysis of data from smart sensors.

We used Kubernetes features to create dynamic disturbances during experiments:

1. Service Node Fluctuation and Restart: This simulates changes in edge node computing power. We randomly trigger Pod rescheduling, which changes service instance IPs and physical locations.
2. Elastic Scaling of Microservice Instances: Based on simulated traffic changes, the number of microservice instances dynamically adjusts between 1 and 5. This makes the service call dependency graph $G_{t}$ change in real time with t. Therefore, ST-GraphRCA must accurately track fault propagation even as the topology changes, with edges $E_{t}$ connecting and disconnecting.

Since edge devices often lack powerful hardware, we did not use GPUs on worker nodes. This tests the lightweight nature of ST-GraphRCA. The Linux Traffic Control tool is used to simulate unstable edge network conditions, such as random delay and packet loss. Detailed hardware and software configurations are shown in Table 1.

To comprehensively evaluate the robustness of the model in heterogeneous IoT scenarios, five datasets (Dataset A@1–A@5) were designed. The key hyper-parameter settings for ST-GraphRCA and the experimental design are summarized in Table 2. These datasets cover a variety of typical edge faults ranging from resource exhaustion to network jitter, as shown in Table 3. Via Chaos Engineering techniques, a total of 500 fault scenarios across four categories were injected, involving 3917 computing tasks.

To validate the effectiveness of ST-GraphRCA in IoT edge environments, ST-GraphRCA was compared against five mainstream root cause analysis methods. The baseline models encompass the latest multi-modal fusion, Graph Neural Networks (GNNs), and Large Language Model (LLM)-driven techniques, as well as classic statistical methods, as described below:

1. Mulan [41]: Mulan is an offline diagnosis method for microservice systems. It uses a log-specific large language model (LLM) to extract semantic features from logs. Through contrastive learning, these features are aligned with structured metric data in a unified latent space. This multimodal integration supports the construction of a service causal graph. However, it incurs greater computational overhead.
2. InstantOps [42]: A defensive operations framework was proposed, which integrates fault prediction with diagnosis through a unified representation method that fuses logs, metrics, and Kubernetes native events. A graph neural network was used to capture service call topology. A gated recurrent unit was used to model time series changes. A permutation test was applied to measure the contribution of each node to faults. The root cause was then located.
3. Chain-of-Event [43]: An event-graph-based reasoning model was proposed. All anomalies in the system were abstracted as fine-grained events. Transition probabilities between events were learned from historical fault data. A weighted event causal graph was built. The diagnosis process of SRE experts was simulated. The most probable fault propagation path was searched for in the graph. Result readability and expert trust were emphasized.
4. OCEAN [44]: A recent online algorithm was designed for real-time data streams. Offline batch processing was not used. Dilated convolutional neural networks were used to capture long-term history. A graph neural network was used to update the causal structure in real time. A multi-factor attention mechanism was introduced. The weights of logs and metrics were adjusted based on data quality. Dynamic causal drift in microservice systems was addressed. This method represents a frontier direction in online diagnosis.
5. Trace Tradition [45]: A set of traditional IT operations methods was defined. These methods were used when system or service faults occurred. Logs were analyzed. System performance was monitored. Configurations were checked. Other related investigation activities were carried out to find the root cause.

### 4.2. Experimental Comparative Analysis

Top-k Accuracy (A@k), F1-Score, and MAR (Mean Average Recall) were adopted as evaluation metrics. Table 4 presents the comprehensive performance of each algorithm across the five datasets. The descriptions of the primary anomaly types injected into the five datasets (Dataset A@1 to A@5) are as follows:

1. Dataset A@1 (Mainly Injects Data Skew): Simulates computation skew problems caused by uneven IoT data distribution. This reflects real scenarios in heterogeneous IoT environments where vast differences in reporting frequencies among different sensor devices lead to severe load imbalances among edge computing nodes.
2. Dataset A@2 (Mainly Injects CPU Resource Exhaustion): Simulates edge gateway overload caused by high-frequency concurrent computing. By injecting infinite loop invocations into Executor containers, this reproduces system paralysis scenarios caused by the exhaustion of computing resources when edge nodes process massive concurrent sensor data.
3. Dataset A@3 (Mainly Injects Application Logic Errors): Simulates edge application logic crashes or data validation failures. By injecting erroneous data substitution at the Job/Application layer, this reproduces task execution failures on the edge side caused by dirty sensor data input or algorithmic logic defects.
4. Dataset A@4 (Mainly Injects Memory Leak/Overflow): Simulates memory overflow caused by sensor data bursts. By injecting list duplication into computing tasks, this reproduces memory crash scenarios in resource-constrained edge devices when coping with sudden data floods.
5. Dataset A@5 (Mainly Injects Network Latency): Simulates inter-device communication jitter in weak-network edge environments. By injecting API delays at the Pod network layer, this reproduces data transmission blocking and timeout issues between edge gateways and terminal devices under unstable network environments (this constitutes the core scenario for validating the DTW temporal alignment capability).

As can be observed from Table 4, ST-GraphRCA achieves an overall F1-Score of 0.89, outperforming other baseline methods. Simultaneously, the model maintains a lower MAR (1.41), demonstrating its robustness across different fault types. Based on Table 4, the limitations of each comparison algorithm in edge environments are analyzed in depth:

1. Mulan: Although it exhibited extremely strong semantic understanding capabilities for logical faults such as A@3 (0.90) by utilizing a Large Language Model (LLM), its performance suffered a severe decline in the weak network environment of A@5 (0.65). This is attributed to its significant offline batch processing latency and computational overhead. Mulan relies on contrastive learning to construct causal graphs; the inference process is time-consuming and requires batch data. Consequently, it cannot achieve real-time response in edge scenarios with frequent network jitter, resulting in a capability to capture transient faults that is substantially weaker than the lightweight ST-GraphRCA.
2. InstantOps: As a predictive model based on GNNs and GRUs, it performs excellently in conventional scenarios such as A@1 (0.95). However, its accuracy in A@5 (0.58) is even lower than that of certain traditional methods. This is attributed to its strong dependency on full Trace data. In edge environments characterized by weak networks and high packet loss, Trace links experience severe breakage, causing the spatial topological structure upon which the GNN relies to fragment. The model fails to effectively aggregate neighbor node information, thereby losing the topological basis for root cause inference.
3. Chain-of-Event (CoE): Its explainable reasoning based on event graphs holds significant advantages in complex business logic faults such as A@3 (0.91). However, it performs mediocrely in terms of overall F1-Score (0.75) and in resource scenarios like A@2 (0.82). The core reasons are its cold start problem and dependency on historical data. Edge environment devices are heterogeneous and fault patterns are variable; it is difficult for CoE to cover all causal patterns using limited historical data. Consequently, when facing unseen resource contention or environmental interference, causal graph paths are missing, making it difficult to localize the root cause.
4. OCEAN: Although OCEAN is designed as an online algorithm and performs excellently in long-term memory leak detection (A@4, 0.92), its accuracy is lower in CPU overload scenarios (A@2, 0.68) due to model complexity and resource contention. The multi-factor attention and dynamic graph updating in OCEAN pose a significant computational burden, constituting a major source of its overhead. In situations where the edge gateway CPU is already overloaded, the complex diagnostic model contends for resources with business processes. This not only causes slow operation but may also render the model completely ineffective due to termination by the system OOM (Out of Memory) Killer.
5. Trace Tradition: The traditional Pearson Correlation Coefficient method achieves an F1-Score of only 0.24. This shows that simple statistical linear correlation analysis fails completely when facing complex non-linear cascading faults in microservices, especially on the edge side where trace data is incomplete.

In summary, we analyzed and compared several baseline methods: Mulan, InstantOps, Chain-of-Event, OCEAN, and Trace Tradition. In cloud data centers, these methods can use LLMs and deep graph learning to achieve high diagnostic accuracy. However, when applied to edge computing, they still face problems. These problems include real-time performance, the need for precise data timing alignment, generalization ability and high resource cost. ST-GraphRCA tackles these challenges. It combines several strategies. First, PCA-DTW solves the problem of timing misalignment in weak networks. Second, it uses the net anomalous outflow within a forward propagation graph to locate the source node. Finally, it applies a High-Utility Mining (HUM) algorithm with reachability constraints. Together, these give a good balance of accuracy, robustness, and efficiency. This balance meets the operational needs of IoT edge systems.

### 4.3. Ablation Study

An ablation study was designed to test the effect of core modules in ST-GraphRCA. Three variant models were built. They were compared with the full model on five datasets.

1. PCA-DTW: used to compute a node deviation from its reference vector. This variant was used to test the need for time-series alignment in weak edge networks.
2. Net-Flow: The flow-conservation based net anomaly outflow metric was removed. Only raw anomaly scores were used to build a forward-propagation graph for ranking. This variant was used to test whether the flow-conservation rule helps distinguish active root causes from passive victims.
3. Topo-Constraint: The topology reachability pruning mask in the mining algorithm was removed. Pattern mining was carried out under an unconstrained, fully connected assumption. This variant was used to test whether causal-subspace constraints help against noise.

**Figure 6:**
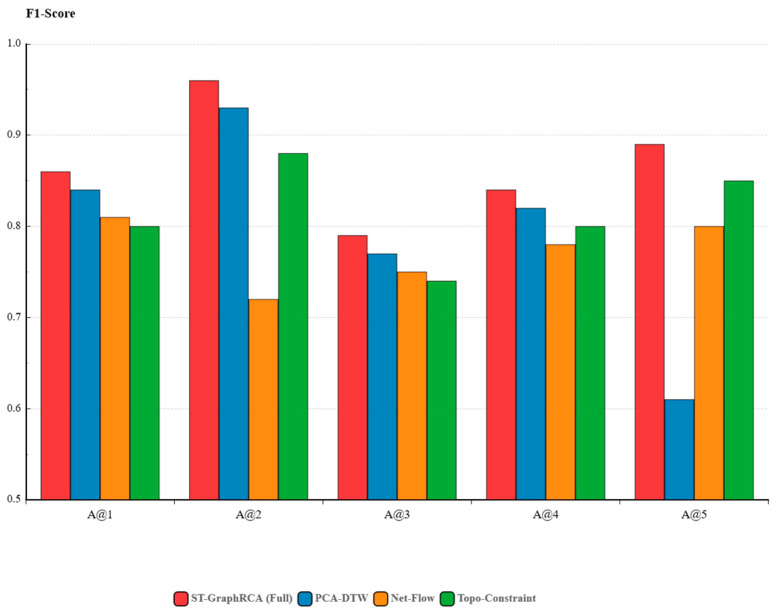
Histogram of ablation experiment.

The results are shown in Table 5 and Figure 6. The full ST-GraphRCA model achieved the best F1-score in all cases. The average value was 0.89. Performance drops of the variants show the role of each module.

1. PCA-DTW is against network jitter: In Dataset A@5 (Network Latency), a clear drop was observed after the PCA-DTW module was removed. The F1-score fell from 0.89 to 0.61. This result shows that random jitter and packet loss exist in edge networks. Standard distance measures cannot handle nonlinear time misalignment. Many false alarms are produced. PCA-DTW restores true data relations through semantic grouping and adaptive alignment.
2. Flow conservation reduces cascade fault errors: Dataset A@2 (CPU Exhaustion) simulates cascade overload caused by high concurrency. After the net outflow mechanism was removed (Net-Flow), performance dropped to an F1-score of 0.72. The main reason was the failure to separate root nodes from victim nodes in cascade faults. True root nodes slow down due to their own compute overload. Downstream nodes remain normal but become blocked while waiting for upstream responses. Both show high anomaly values in metrics. Without flow direction, root sources and passive victims cannot be separated. Many victim nodes are ranked as root causes. After flow conservation was added, net outflow values enabled clear separation.
3. Topology constraints improve noise resistance: The Topo-Constraint variant showed acceptable results in simple cases such as A@1. However, the overall average score was lower than the full model (0.82 vs. 0.89). Without topology reachability limits, unrelated nodes were grouped into the same fault pattern. Non-causal noise was introduced. Top-k ranking accuracy was reduced.

### 4.4. Efficiency Analysis

Average root cause time per fault was measured on edge devices. All methods were compared. Time was reported in ms.

**Figure 7:**
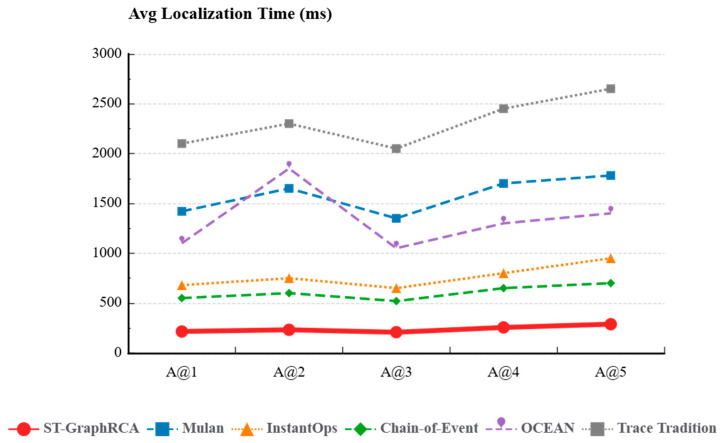
Root cause localization performance line chart.

Figure 7 highlights the substantial performance edge of ST-GraphRCA across all fault scenarios. With a stable average latency of 238.8 ms, the model improves processing speed by 6.4× and 9.6× compared to the complex LLM-based Mulan (1544.0 ms) and traditional Trace methods (2290.0 ms), respectively. This efficiency fully meets the stringent real-time requirements of Industrial IoT (IIoT) systems. In contrast, while the 2025 baseline algorithms achieve high accuracy, they incur varying degrees of latency penalties:

1. Mulan: Latencies consistently exceed 1500 ms, a limitation inherent to its offline batch processing architecture. The computational burden of log embedding and contrastive learning in Large Language Models (LLMs) creates significant inference delays, rendering the model unsuitable for millisecond-level real-time demands.
2. OCEAN: Despite being an online algorithm, OCEAN experiences a sharp latency spike to 1850 ms during CPU Overload (A@2) scenarios. This corroborates earlier analysis: on edge gateways with limited hardware, OCEAN’s complex dilated convolutions and multi-factor attention mechanisms trigger severe resource contention. Consequently, the diagnostic process itself stalls, leading to a drastic drop in throughput.
3. InstantOps & Chain-of-Event: These models fall into the intermediate range of 600–900 ms. While faster than legacy trace methods, they remain 2–3× slower than ST-GraphRCA due to the computational overhead associated with GNN graph traversal and event chain searching.
4. Trace Tradition: Exhibiting the highest latency (>2000 ms), this approach scales linearly with fault complexity; effectively confirming that full trace aggregation is computationally infeasible at the edge.

By leveraging the lightweight PCA-DTW algorithm, ST-GraphRCA successfully constrains computational complexity to a linear level. It stands as the sole model capable of maintaining sub-300 ms responsiveness under the dual constraints of weak edge networks and resource overload (A@2, A@5).

### 4.5. Parameter Sensitivity Analysis

#### 4.5.1. Impact of DTW Penalty Factors on Performance

The dynamic penalty strategy is the core of PCA-DTW module. To verify the sensitivity, we varied the change rate of the DTW penalty factor. The first step is to define a multiplier λ. This multiplier dynamically adjusts the insertion and deletion penalty factors, β and γ. Their variation values are $△\beta_{low}$ and $△\gamma_{low}$, which can be written as $△{\gamma_{low}}^{t}=\lambda \cdot △\gamma_{low}$ and $△{\beta_{low}}^{t}=\lambda \cdot △\beta_{low}$. The second step is to change these values within a set interval and observed how the F1-score changed. Then the dataset A@5 is used for the test. This dataset contains complex mixed noise and nonlinear coupling between network jitter and application-layer metrics. In the network delay scenario, the network penalty factor γ. is adjusted. This produced a curve showing the change in the F1-score.

**Figure 8:**
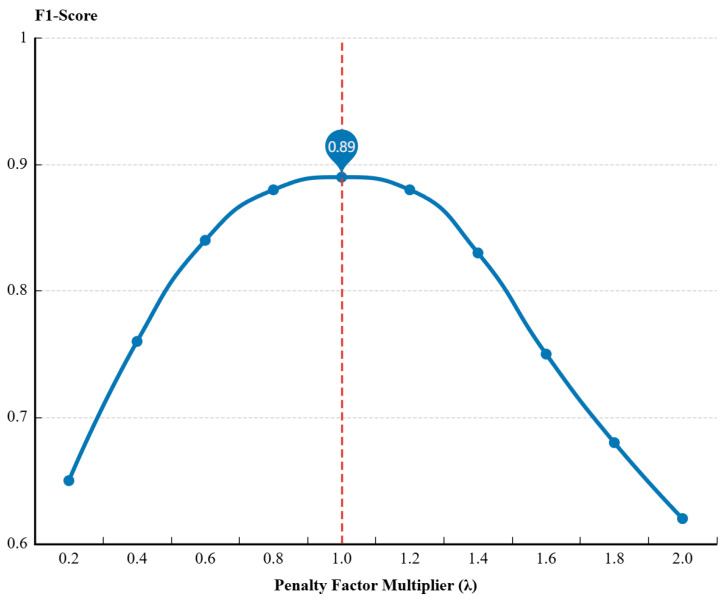
Sensitivity of DTW Penalty Factors (The red dashed line indicates the optimal parameter setting where the model achieves the best performance.).

As shown in Figure 8, the model achieves optimal performance when λ≈1.0. When λ<0.6, ${\beta_{low}}^{t}$ decreases, the F1-score drops significantly. This is because an excessively low change value ${\gamma_{low}}^{t}$ of the network penalty factor γ causes the DTW algorithm to tend toward linear matching. The algorithm cannot handle real-time offsets. This leads to false negative results. In contrast, the algorithm performance degrades when λ>1.6, ${\gamma_{low}}^{t}$ increases. An excessively large change value ${\gamma_{low}}^{t}$ of the network penalty factor γ is the reason for this decline: the penalty makes the DTW algorithm overly flexible, which causes the alignment of irrelevant noise patterns and further generates false positive results.

In summary, the stability experiment of the F1-score within the range of [0.8, 1.2] shows that the parameter setting of λ≈1.0 is robust and not sensitive to minor fluctuations.

#### 4.5.2. Impact of Graph Thresholds on Performance

**Figure 9:**
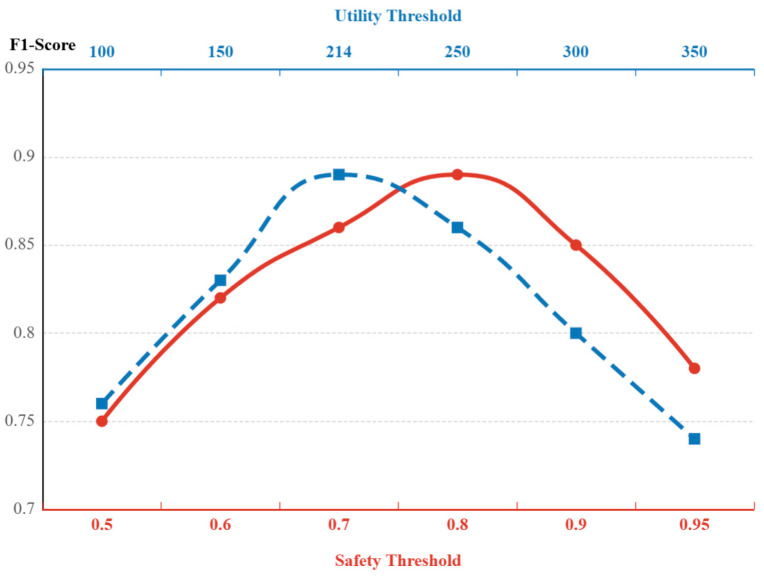
Sensitivity of Graph Thresholds (The figure illustrates the impact of varying threshold parameters on the model’s performance. The red solid line with circular markers indicates the performance trend across different Safety Thresholds. The blue dashed line with square markers shows the performance trend across different Utility Thresholds. The markers represent the specific threshold values evaluated.).

To evaluate the model stability under different filtering strengths, the two parameters are tested. These are the Net Outflow Safety Threshold τ and the Preset Utility Pruning Threshold δ. We analyzed their impact on root cause localization performance. The results are shown in Figure 9.

1. Impact of the Safety Threshold. The red solid line (lower x-axis) shows the trend of τ. The model’s F1-Score is best (0.89) when τ is set to 0.8. When τ is less than 0.7, the model cannot distinguish the difference between active fault sources and passive victims. Both have positive outflow in this case. This leads to a higher false positive rate. When τ is greater than 0.9, the judgment is too strict. Some weaker, early-stage faults are missed. The experiment shows that setting τ around 0.8 can better separate active fault sources from passive victims.
2. Impact of the Preset Utility Threshold δ. The blue dashed line (upper x-axis) shows the effect of changing the Preset Threshold δ in high-utility mining from 100 to 350. The F1-Score peaks when δ is 214. If the threshold is too low (δ < 150), pruning is not strong enough. Many low-utility noise items (like occasional non-causal calls) remain in the candidate set. This disturbs the ranking of the Top-k root causes, so precision drops. If the threshold is too high (δ > 250), pruning is too aggressive. Some real fault patterns that are low-frequency but high-risk are wrongly removed. This causes a clear drop in recall. The experiment shows that setting (δ) around 214 better balances pruning efficiency and the retention of fault patterns.

In summary, ST-GraphRCA shows high stability within the recommended parameter ranges of [0.75, 0.85] (τ) and [200, 230] (δ). This shows the model robustness to parameter settings.

#### 4.5.3. Impact of PCA Component Selection (k) on Performance

**Figure 10:**
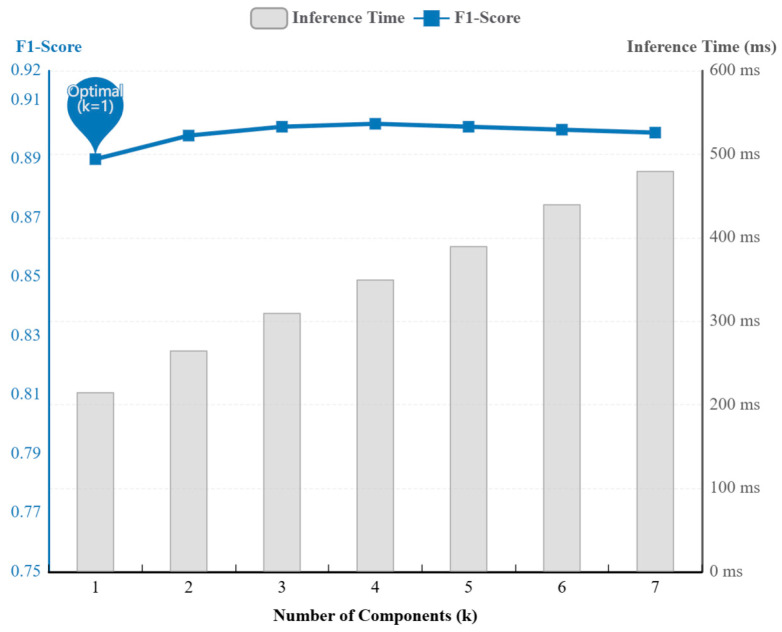
Impact of PCA Component Selection (k).

To find the best balance between detection accuracy and computational efficiency for resource-limited edge nodes, we evaluated how the number of principal components, k, affects model performance. The results are shown in Figure 10.

The first step is to perform a stress test on the complex Dataset A@5. The second step is to teste *k* values from 1 to 7. This range covers all seven original feature dimensions, including network metrics (throughput, RTT, retransmission rate) and computational resource metrics (CPU usage, memory usage, deadlock count, I/O wait).

The results are shown in Figure 10. The analysis is detailed below.

Phase k=1→4: With k = 1, the model’s F1-Score was already 0.89. This means the first principal component successfully captured the main anomaly information. When k increased to 4, the F1-Score rose only slightly to a peak of 0.902. This is an improvement of less than 1.4%. This result supports the statistical signal processing theory mentioned before. It shows that most useful variance is in the first component.

Phase k=5→7: When k was increased further to 5, 6, and 7, the F1-Score did not go up. It even dropped a little to 0.899. This indicates that higher-order components mainly contain random environmental noise, such as sensor errors. Adding them actually harms the clarity of the fault features.

Computational Cost Evaluation: Inference time increased significantly as k increased. For example, choosing k = 4 for a small accuracy gain makes the inference delay jump from 215 ms to 350 ms. This is a 63% increase. Memory use also went up noticeably.

In summary, we balanced accuracy and efficiency for edge devices. Since k = 1 gives near-optimal accuracy (98.7% of the peak) at the lowest computational cost, we set k = 1 as the best configuration for ST-GraphRCA.

## 5. Deployment Analysis for Application Scenarios

To better illustrate how ST-GraphRCA can be applied in real-world settings, this section analyzes its deployment in several typical scenarios.

1. Industrial IoT and Manufacturing Gateways: In smart factory production lines, edge gateways need to process real-time data from many PLCs and sensors. When a fault happens due to network issues or overload, ST-GraphRCA can be deployed directly on industrial edge gateways. Its low inference delay lets it quickly find the root cause before the fault spreads. This helps reduce unplanned downtime and keeps production running.
2. Smart City Traffic Sensing Networks: In smart traffic systems, roadside units analyze vehicle and infrastructure data in real time. Urban edge networks often have random delays and packet loss. ST-GraphRCA uses its PCA-DTW elastic alignment to handle timing sync problems well. This allows accurate fault tracing across different monitoring nodes. As a result, traffic signal control systems remain stable.
3. Remote Environment Monitoring and Resource-Limited Gateways: For sensor networks in remote or power-limited areas, ST-GraphRCA is very lightweight. It needs only low performance to run. Relying mainly on the first principal component keeps its computation simple. This makes it possible to embed the model into tiny edge terminals with very few resources. These terminals can then perform local, self-contained anomaly diagnosis.

## 6. Conclusions

The ST-GraphRCA model proposed herein addresses the critical issue of anomaly fault localization in edge network environments. In the anomaly feature extraction phase, PCA is employed to identify the factors disrupting the alignment of multi-source O&M data. Then, the alignment strategies of the DTW method are dynamically adjusted based on different influence types. By this approach, the temporal misalignment caused by network uncertainties in edge environments is effectively overcome, significantly enhancing the accuracy of anomaly feature extraction. Within the fault propagation chain, graph structures and network flow conservation theory are leveraged to implement causal inference, quantitatively determining candidate solutions for anomaly root causes. Finally, an efficient root cause localization mining algorithm is designed, which utilizes pruning strategies to rapidly screen for low-frequency, high-risk anomaly root causes. The proposed model possesses distinct advantages regarding lightweight operation and robustness in anomaly fault localization.

Future work will be directed towards further optimizing computational demands to compress the algorithm for execution on micro-terminals such as sensors. Additionally, the integration of Federated Learning is planned to enable collaborative training without transmitting raw data, thereby enhancing data security.

## Figures and Tables

**Table 1:** Experimental Environment Configuration.

Category	Item	Specification
Hardware	Master Node (Edge Server)	Intel Xeon Gold 6248R @ 3.00 GHz, 64 GB RAM, 1 TB SSD
	Worker Nodes (×32)	Simulated via KVM: 2 vCPU, 4 GB RAM, 50 GB Disk
	Network	1 Gbps LAN, bandwidth restricted to 100 Mbps via tc to mimic edge constraints
Software	Operating System	Ubuntu 20.04.6 LTS (Kernel 5.4.0)
	Orchestration	Kubernetes v1.24.0, Docker v20.10.12
	Monitoring	Prometheus v2.35 (Metric Collection), Jaeger v1.34 (Trace Collection)
Implementation	Programming Language	Python 3.9.12
	Key Libraries	scikit-learn 1.1.1 (PCA), dtaidistance 2.3.10 (DTW), networkx 2.8 (Graph), numpy 1.21.5

**Table 2:** Key Hyper-parameter Settings of ST-GraphRCA.

Module	Parameter	Symbol	Value	Description
3.2	Sliding Window Size	t	60 s	Time steps for the sliding window.
	Penalty Factor (Insert)	γ	0.45	Init γ=1, Reduced penalty for network-induced lag $△\gamma_{low}=0.45$.
	Penalty Factor (Delete)	β	0.57	Init β=1, Reduced penalty for computation-induced loss $△\beta_{low}=0.57$.
	PCA Component	*k*	1	Number of principal components retained.
3.3	Smoothing Factor	ψ	0.02	Exponential moving average factor for weight updates.
	Safety Threshold	τ	0.8	Threshold for identifying active root causes.
	Abnormal Threshold	φ	0.42	Anomalies spreading downstream.
3.4	Minimum External Utility Threshold	ρ	0.05	Preventing excessively small utility values and suppressing the interference of non causal noise.
	Utility Threshold	δ	214	Preset utility threshold.
Baselines	Learning Rate	lr	1 × 10^−3^	Applied to DL-based baselines (Mulan, InstantOps, OCEAN).
	Batch Size	B	32	Mini-batch size used for training baseline models.
	Training Epochs	E	100	Maximum training epochs with early stopping for baselines.

**Table 3:** Fault injection strategies in edge computing scenario.

Fault Type	Injection Target	Implementation Method	IoT Scenario Mapping
CPU Exhaustion	Executor Container	Infinite loop invocation	Edge gateway overload caused by high-frequency concurrent computing
Memory Leak	Computing Task	List duplication	Sensor data bursts leading to Out of Memory (OOM)
Network Latency	PodNetwork Layer	API delay	Inter-device communication jitter under weak network conditions
Logic Error	Job/Application	Erroneous data substitution	Edge application logic crash or data validation failure

**Table 4:** RCA Performance Comparison (Top-1 Accuracy & Overall Metrics).

Algorithm	A@1	A@2	A@3	A@4	A@5	F1-Score	MAR
ST-GraphRCA	0.86	0.96	0.79	0.84	0.89	0.89	1.41
Mulan	0.92	0.88	0.9	0.82	0.65	0.81	1.55
InstantOps	0.95	0.91	0.85	0.89	0.58	0.78	1.82
Chain-of-Event	0.88	0.82	0.91	0.76	0.72	0.75	1.95
OCEAN	0.94	0.68	0.88	0.92	0.85	0.72	2.10
Trace Tradition	0.35	0.45	0.25	0.35	0.55	0.24	4.55

**Table 5:** Results of ablation experiments on different datasets.

Algorithm	A@1	A@2	A@3	A@4	A@5	F1-Score
ST-GraphRCA	0.86	0.96	0.79	0.84	0.89	0.89
PCA-DTW	0.84	0.93	0.77	0.82	0.61 ⬇	0.79
Net-Flow	0.81	0.72 ⬇	0.75	0.78	0.80	0.77
Topo-Constraint	0.80	0.88	0.74	0.80	0.85	0.82

The downward arrow (⬇) indicates a performance decrease compared to the full model when a specific module is removed or replaced.

## Data Availability

The raw data supporting the conclusions of this article will be made available by the corresponding authors on request.
